# Serum urate and lung cancer: a cohort study and Mendelian randomization using UK Biobank

**DOI:** 10.1186/s12931-021-01768-y

**Published:** 2021-06-16

**Authors:** Laura J. Horsfall, Ian P. Hall, Irwin Nazareth

**Affiliations:** 1 Research Department of Primary Care and Population Health, University College London, Royal Free Hospital Campus, London, NW3 2PF UK; 2 University of Nottingham, 6123, Division of Respiratory Medicine, Nottingham, Nottinghamshire, UK; 3 National Institute for Health Research Nottingham BRC, Nottingham, UK

**Keywords:** Mendelian randomization, Urate, Lung function, Causal relationship, Lung cancer, Smoking phenotypes

## Abstract

**Background:**

Serum urate is the most abundant small molecule with antioxidant properties found in blood and the epithelial lining fluid of the respiratory system. Moderately raised serum urate is associated with lower rates of lung cancer and COPD in smokers but whether these relationships reflect antioxidant properties or residual confounding is unknown.

**Methods:**

We investigated the observational and potentially causal associations of serum urate with lung cancer incidence and FEV_1_ using one-sample Mendelian randomization (MR) and the UK Biobank resource. Incident lung cancer events were identified from national cancer registries as FEV_1_ was measured at baseline. Observational and genetically instrumented incidence rate ratios (IRRs) and risk differences per 10,000 person-years (PYs) by smoking status were estimated.

**Results:**

The analysis included 359,192 participants and 1,924 lung cancer events. The associations between measured urate levels and lung cancer were broadly U-shaped but varied by sex at birth with the strongest associations in current smoking men. After adjustment for confounding variables, current smoking men with low serum urate (100 µmol/L) had the highest predicted lung cancer incidence at 125/10,000 PY (95%CI 56–170/10,000 PY) compared with 45/10,000 PY (95%CI 38–47/10,000 PY) for those with the median level (300 µmol/L). Raised measured urate was associated with a lower baseline FEV_1_. The MR results did not support a causal relationship between serum urate and lung cancer or FEV_1_.

**Conclusions:**

We found no evidence that serum urate is a modifiable risk factor for respiratory health or lung cancer.

**Supplementary Information:**

The online version contains supplementary material available at 10.1186/s12931-021-01768-y.

## Background

Purine compounds, including adenine and guanine, are essential for many cell processes. Although it is possible to synthesise purines, animals derive large quantities from food, including certain meats and seafood. Excess dietary purines are transported in blood to the liver where they are converted (oxidised) to urea and allantoin by the enzyme uricase. However, uricase is absent in humans due to loss-of-function mutations in the uricase gene [1], which means urate levels in human blood are around fifty times higher than non-primate mammals [2]. For some people, a purine-rich diet can lead to the condition gout, where urate crystals accumulate in joints causing pain and inflammation. However, the reduction and loss of uricase activity during higher primate evolution together with some unusual features of urate metabolism, has led to speculation that raised levels may also benefit humans physiologically [3–5].

Serum urate has powerful antioxidant properties in vitro and, with an average concentration of 300 µmol/L, it is the most abundant molecule with antioxidant properties present in human blood [6, 7]. Estimates suggest as much as 50% of human blood antioxidant capacity is accounted for by the action of serum urate [8]. This has led to theories that the low cancer rates and longevity in hominids relative to other mammals are partly due to the reduction and loss of uricase activity [9].

As well as being found at high concentrations in blood, urate is found at high concentrations in human respiratory tissues and the epithelial lining fluid of the respiratory tract and could provide an important first-line defence against environmental oxidants from smoke and pollution [10, 11]. Our group’s previous large-scale cohort study of people living in the UK found that cigarette smokers with moderately high serum urate had substantially lower rates of COPD and lung cancer [12]. However, the cohort only included people with a urate measure in their primary health care records, which is a highly selective sample and replication in an independent cohort is warranted. Furthermore, the association may reflect residual confounding or reverse causation rather than antioxidant properties of urate.

## Methods

### Aims

The first aim of this study was to see whether we could replicate the association between measured serum urate and respiratory outcomes. The second aim was to examine whether there was any evidence supporting a causal association with genetically predicted serum urate and respiratory cancer(Mendelian randomization). We also examined the associations between urate and respiratory function as a phenotype that might also be influenced by endogenous antioxidant activity.

### Data source

We used The UK Biobank Resource, a prospective cohort study of over 500,000 participants aged 40–69 years, recruited between 2006 and 2010 from around the UK [13]. Further information on UK Biobank such as the processing of biological samples including DNA is available at the following: https://www.ukbiobank.ac.uk/. The quality control and imputation of SNPs, indels and structural variants are reported elsewhere [14].

### Study design

The methods we report are similar to our earlier study on serum bilirubin using UK Biobank [15]. In brief, we analysed the longitudinal relationship between serum urate levels and lung cancer and the cross-sectional relationship between serum urate and forced expiratory volume in 1 s (FEV_1_). We estimated the causal relationships between urate levels and these outcomes by applying Mendelian randomization (MR) to individual-level data. The protocol was approved by UK Biobank in July 2018 (ID:5167) and we checked the adequacy of sample size using online tools (http://cnsgenomics.com/shiny/mRnd/).

### Inclusion/exclusion criteria

We excluded people who no longer wished to participate in UK Biobank up to August 2020 and applied several genetic exclusions including outliers for genotype missingness or excess heterozygosity, sex aneuploidy and sex discordance (n = 2200). We used a published algorithm to retain unrelated participants [16] (n = 39,642) and finally restricted the sample to “white British” participants based on self-reported ethnic identity and principal components available in the dataset (n = 88,341) [14]. We set the cohort start date at the date when the participant attended the research centre and the exit date was the earliest date of lung cancer diagnosis, loss to follow-up, death or end of the follow-up period. At the time of analysis, the most recent date for complete follow-up for incident cancers was March 2016 for England and Wales and October 2015 for Scotland. Prevalent lung cancer cases were excluded (n = 512).

### Exposures

Almost all participants provided blood samples at the initial assessment centre visit. Serum urate was assayed in theses samples by Uricase PAP (Beckman Coulter AU5800). We selected 31 SNPs for estimating genetically predicted urate levels based on the results of a large-scale Genome Wide Association Analysis (GWAS) of European people [17]. The two lead GWAS SNPs (rs12498742 and rs2231142) are located in renal and gut urate transporters [18] and we analysed these separately as well as in combination with the 28 weaker variants.

### Outcomes

The primary outcome was a new lung cancer diagnosis recorded after study recruitment. Cancer diagnoses in UK Biobank are provided by the NHS Central Register for participants living in Scotland and the Health & Social Care Information Centre for participants living in England and Wales. Diagnoses are coded using the International Classification of Disease (ICD) version 9 and 10 and we selected malignant neoplasms of the trachea and bronchus (ICD10: C33-C34) as the cancers where smoking has the strongest pathophysiological role and highest attributable risk [19]. In addition to the national cancer registries, we used self-reported cancer diagnosis to identify prevalent cancers.

We also examined the relationship with FEV_1_. Spirometry was performed during the baseline assessment using a Vitalograph Pneumotrac 6800. The participant was asked to record two to three blows (lasting for at least 6 s) within approximately six minutes. We used the maximum value of FEV_1_ meeting the assessor’s acceptability criteria. Further details on spirometry are reported elsewhere [20].

### Other variables

We included important predictors of lung cancer in analyses including age, calendar year, genetic sex, population sub-structure (first 40 principal components) recruitment centre, height, weight and self-reported smoking status [21, 22]. Weight is strongly associated with urate levels and there is evidence that weight is causally associated with lung cancer [23]. Further, the lead GWAS SNP (rs12498742) is located in a gene that has a role in glucose homeostasis (*SLC2A9*) that could potentially influence weight. Therefore, we examined models with and without this variable. In a subset of people with a history of regular smoking, we further adjusted for waist circumference, exposure to smoke at home, Townsend social deprivation index, antioxidant supplements, alcohol intake and nitrogen dioxide air pollution.

As a supplementary analysis, we also examined whether participants genetically predisposed to raised urate were less likely to have a positive family history of lung cancer and a lower baseline prevalence of chronic obstructive pulmonary disease (COPD).

### Interactions

We fitted models separately for men and women given the different levels average urate levels as well as evidence of differential genetic effects of SNPs on urate levels [24]. We previously reported strong interactions between urate and smoking status with no clear association in non-smokers but strong negative associations in current smokers [12]. We therefore estimated associations by self-reported smoking status (never, former and current) and smoking intensity (1–19 cigarettes per day or 20 or more cigarettes per day) by including multiplicative interaction terms in the models for each sex. Pack-years of smoking was available for a subset of participants and we described continuous-by-continuous interactions with urate.

### Statistical analyses

Serum urate levels were divided into sex-specific quintile categories to describe the univariable associations with other covariates. We identified and excluded outlier values for continuous variables using multivariate approach (blocked adaptive computationally efficient outlier nominators algorithm) including age and sex with a 15% threshold of the chi-squared distribution [25]. To estimate the observational incidence rate ratios (IRRs) per 100 μmol/L increase in serum urate, we used multivariable Poisson regression with robust standard errors and age as the time scale. We explored non-linear relationships by applying restricted cubic spline-interpolation using Harrell’s default percentiles and selecting the transformation that minimised the Akaike and Bayesian information criteria (AIC/BIC). To easily visualise non-linear transformations and interactions, we calculated the margins of response as adjusted incidence rates at different levels of urate while holding all other variables at their observed values. We applied a user-written programme for data visualisation [26] and standard errors for marginal effects were calculated using the delta method. We checked for proportionality of associations with the time scale by testing interaction terms. Continuous variables were parameterised as linear and Wald tests were used for calculating p-values for categorical variables and spline transformations. To investigate any reverse causation, we compared the associations after setting the cohort entry date to one and two years after the urate test date.

We estimated the IRRs for lung cancer per 100 μmol/L increase genetically predicted urate using one-sample MR and the two‐stage predictor substitution (2SPS) method [21]. Odds ratios for the supplementary outcomes COPD and family history of lung cancer were also estimated using this method. We used a similar approach, the two stage least squares method (2SLS), to estimate the causal cross-sectional relationship per 100 μmol/L increase genetically predicted urate and FEV_1_ [21]. FEV_1_ was missing not at random for approximately 25% of participants and we used inverse probability weighting in an attempt to reduce the impact of any selection bias. After applying the ERS/ATS criteria for FEV_1_ reproducibility, FEV_1_ was missing for 50% of smokers and we decided against this analysis.

Relatives were excluded using an algorithm in R (v.3.5.1) [16] and all other analyses were done using Stata v.16.1 (Stata Corporation, College Station, Texas).

## Results

Serum urate levels were available for 359,192 participants (Table 1). There were 1924 incident cases of lung cancer diagnosed after recruitment, 15,335 deaths from any cause and 766 participants were lost to follow-up for reasons including emigration. Median follow-up time was 7-years (interquartile range 6–8 years). Men and women with high urate levels were heavier, shorter, more likely to live in socially deprived areas (Table 1). Those in the highest quintile were also more likely to report a clinical diagnosis of lung cancer/COPD and emphysema prior to recruitment (Table 1). However, there were fewer current smokers in the highest quintile. Unadjusted lung function tended to decline as urate increased across smoking categories and the relationship with lung cancer was U-shaped (Table 2).

**Table 1:** Baseline characteristics of UK Biobank participants by sex-specific quintiles of serum urate

	Total	Quintile of serum urate (µmol/L)				
Men		89–294	295–332	333–367	368–411	411–600
Women		89–215	216–248	249–279	280–321	322–600
	N = 359,192	N = 71,716	N = 71,889	N = 71,745	N = 71,927	N = 71,915
Sex	166,618 (46.4%)	33,296 (46.4%)	33,320 (46.3%)	33,278 (46.4%)	33,380 (46.4%)	33,344 (46.4%)
Age at recruitment (IQR)	58.9 (51.3–64.0)	57.3 (49.4–63.1)	58.1 (50.3–63.5)	58.7 (51.3–63.8)	59.5 (52.3–64.2)	60.5 (53.7–64.9)
Weight (kg)	78.3 (15.8)	72.3 (14.0)	75.1 (14.5)	77.7 (14.9)	80.6 (15.5)	85.8 (16.5)
Height (cm)	168.8 (9.2)	169.0 (9.0)	169.0 (9.1)	168.9 (9.2)	168.8 (9.3)	168.4 (9.4)
BMI	27.4 (4.7)	25.2 (3.9)	26.2 (4.0)	27.1 (4.2)	28.2 (4.6)	30.2 (5.2)
Smoking status						
Never	195,303 (54.4%)	41,107 (57.3%)	40,526 (56.4%)	39,585 (55.2%)	38,386 (53.4%)	35,699 (49.6%)
Former	126,440 (35.2%)	21,763 (30.3%)	23,462 (32.6%)	24,988 (34.8%)	26,581 (37.0%)	29,646 (41.2%)
Current	36,227 (10.1%)	8622 (12.0%)	7654 (10.6%)	6951 (9.7%)	6726 (9.4%)	6274 (8.7%)
Missing	1222 (0.3%)	224 (0.3%)	247 (0.3%)	221 (0.3%)	234 (0.3%)	296 (0.4%)
Pack years of smoking (IQR)*	19.5 (10.0–32.5)	18.8 (9.3–31.5)	18.0 (9.0–30.6)	18.8 (9.5–31.5)	19.5 (10.1–32.5)	22.0 (12.0–35.5)
History of lung cancer	528 (0.15%)	101 (0.14%)	82 (0.11%)	98 (0.14%)	91 (0.13%)	156 (0.22%)
Family history of lung cancer	46,291 (12.9%)	8547 (11.9%)	8738 (12.2%)	9218 (12.8%)	9596 (13.3%)	10,192 (14.2%)
History of COPD/emphysema	8167 (2.3%)	1486 (2.1%)	1458 (2.0%)	1515 (2.1%)	1646 (2.3%)	2062 (2.9%)

All continuous variables are mean values with ± 1 standard or medians for skewed data if interquartile ranges (IQRs) are specified

*Previously calculated for 109,312 participants reporting to regularly smoke at least one cigarette/day and who also reported smoking duration

**Table 2:** Mean baseline FEV1 and lung cancer incidence by sex-stratified quintiles categories of serum urate

Smoking status	Sex stratified quintile of urate*	Number	Mean FEV_1_ (SD)	Lung cancer events	Person years (× 10,000 PYs)	Lung cancer incidence rate per 10,000 PYs (95%CI)
Overall	1	71,720	2.93 (0.76)	398	50.4	7.9 (7.2–8.7)
	2	71,895	2.90 (0.77)	366	50.4	7.3 (6.6–8.0)
	3	71,753	2.87 (0.78)	325	50.6	6.4 (5.8–7.2)
	4	71,932	2.83 (0.78)	353	50.6	7.0 (6.3–7.7)
	5	71,952	2.73 (0.79)	482	50.6	9.5 (8.7–10.4)
Never	1	41,109	2.96 (0.75)	48	29.1	1.7 (1.2–2.2)
	2	40,528	2.94 (0.77)	52	28.6	1.8 (1.4–2.4)
	3	39,586	2.90 (0.78)	51	28.0	1.8 (1.4–2.4)
	4	38,387	2.85 (0.78)	56	27.2	2.1 (1.6–2.7)
	5	35,720	2.76 (0.79)	56	25.2	2.2 (1.7–2.9)
Former	1	21,764	2.92 (0.75)	146	15.2	9.6 (8.2–11.3)
	2	23,463	2.89 (0.75)	139	16.4	8.5 (7.2–10.0)
	3	24,993	2.87 (0.76)	142	17.5	8.1 (6.9–9.5)
	4	26,584	2.82 (0.77)	178	18.6	9.6 (8.3–11.1)
	5	29,658	2.71 (0.78)	288	20.7	13.9 (12.4–15.6)
Current	1	8623	2.82 (0.82)	204	6.0	34.0 (29.6–39.0)
	2	7656	2.79 (0.82)	174	5.3	32.8 (28.3–38.0)
	3	6953	2.77 (0.83)	130	4.9	26.7 (22.5–31.7)
	4	6727	2.76 (0.84)	118	4.7	25.2 (21.0–30.2)
	5	6278	2.66 (0.84)	133	4.4	30.3 (25.6–35.9)

*See Table 1 for µmol/L values

**Fig. 1:**
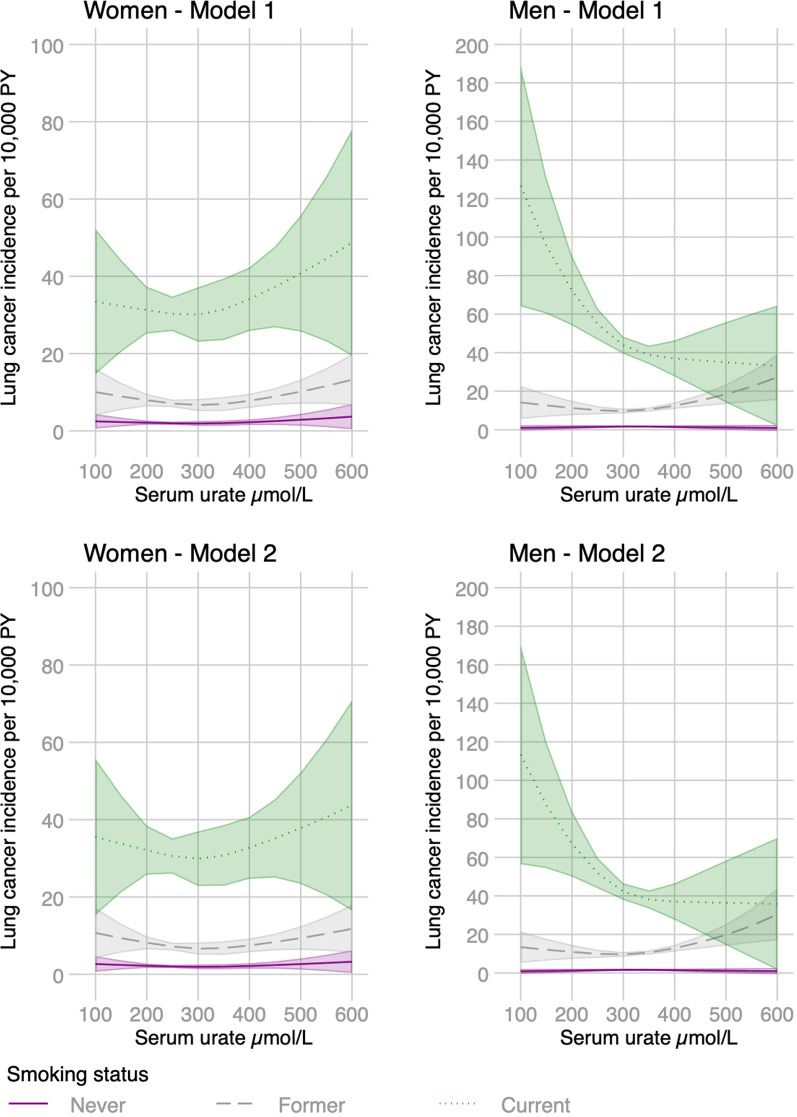
Observational associations between serum urate and lung cancer incidence by sex adjusted for age, calendar year, ethnicity (first 40 principal components), height and recruitment centre (Model 1) and additionally for weight (Model 2). Non-linear relationships were captured using restricted cubic spline transformation with three knots placed at the 10th, 50th and 90th percentiles of urate levels

**Fig. 2:**
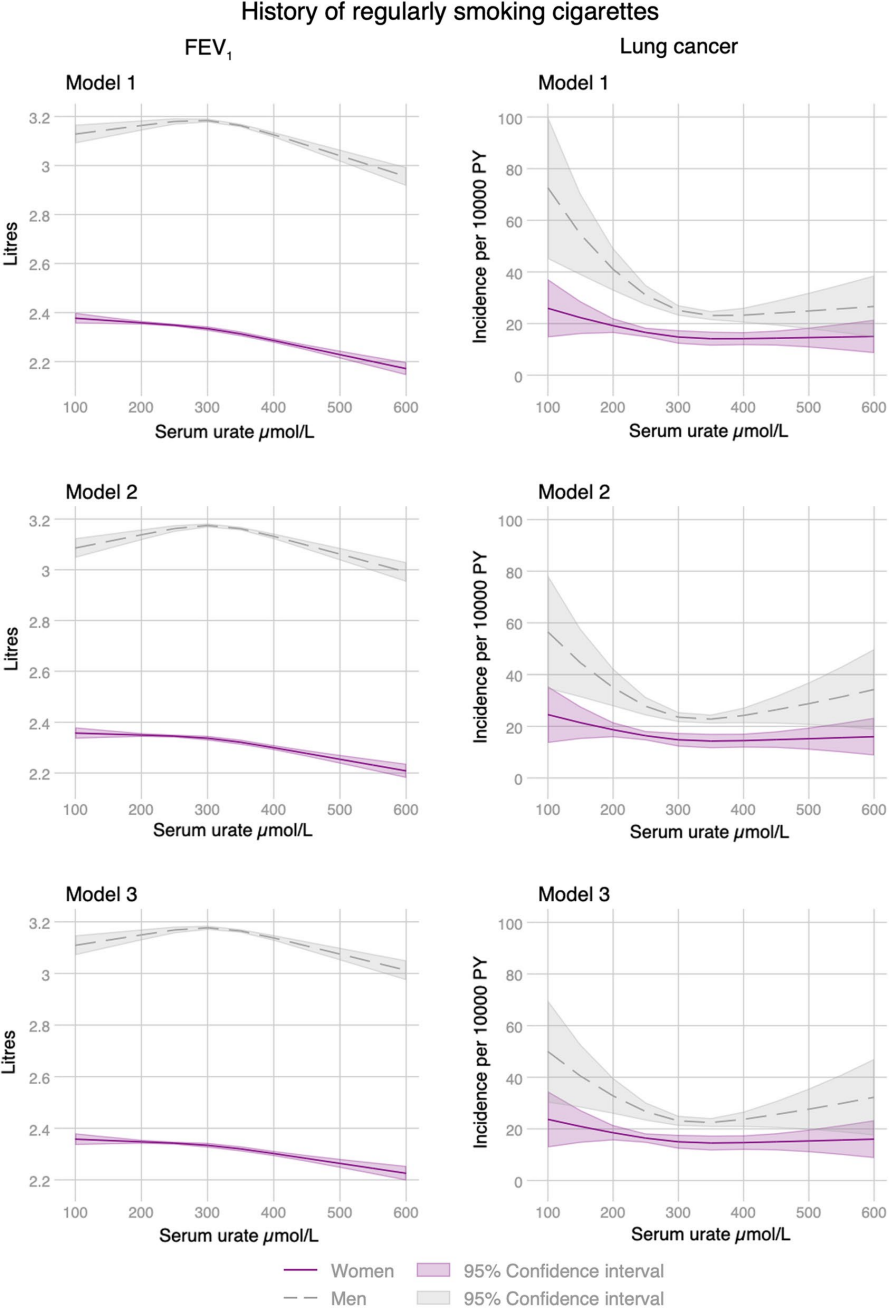
Observational associations of serum urate with FEV_1_ and lung cancer incidence in regular smokers by sex adjusted for age, calendar year, ethnicity (first 40 principal components), packyears of smoking, height and recruitment centre (Model 1), additionally for weight (Model 2) and waist circumference, alcohol consumption, exposure to smoke at home, social deprivation, air pollution levels (nitrogen dioxide), and intake of antioxidant supplements (Model 3). Non-linear relationships were captured using restricted cubic spline transformation with three knots placed at the 10th, 50th and 90th percentiles of urate level

**Fig. 3:**
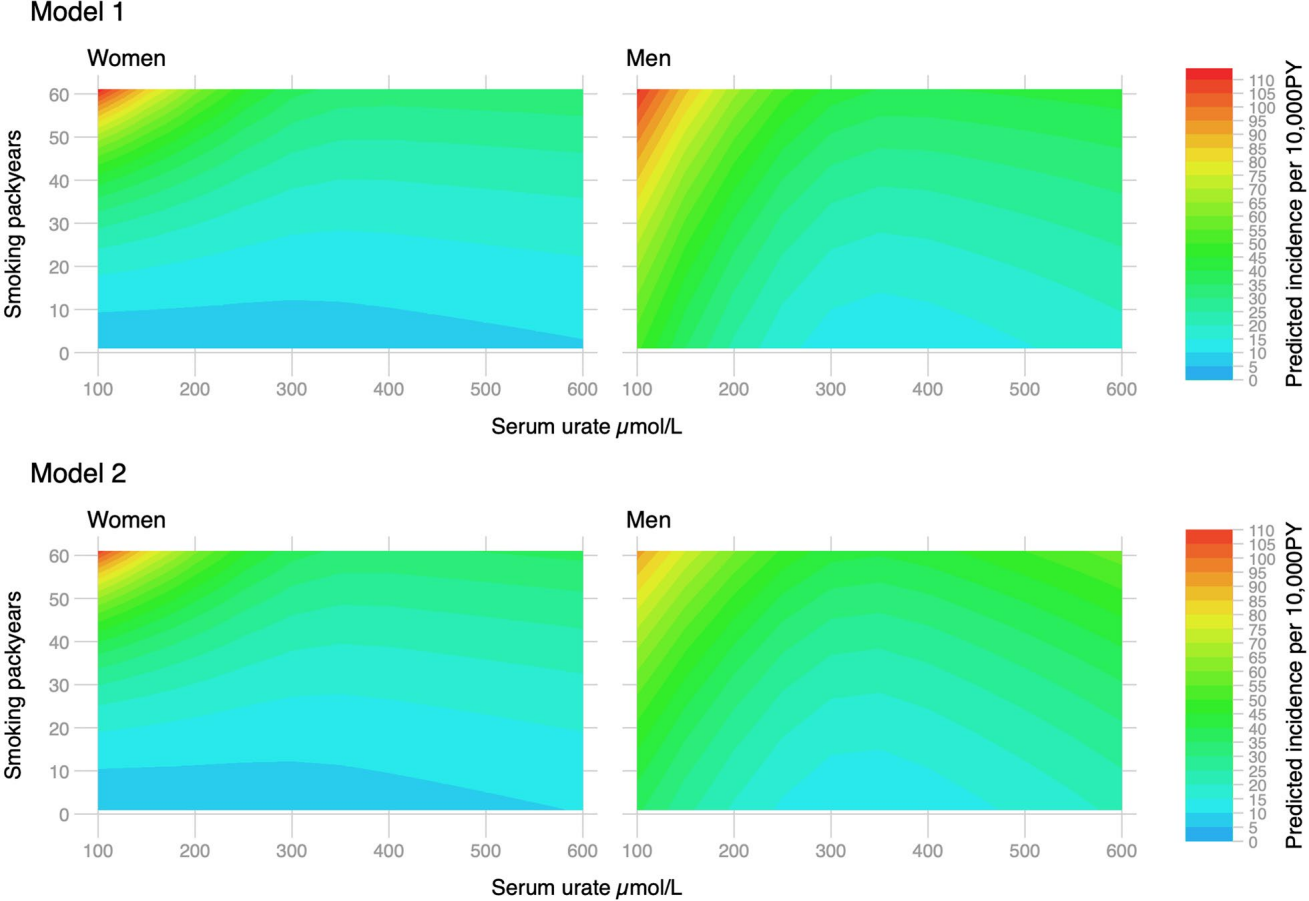
Observational associations between serum urate and lung cancer incidence with interactions with smoking packyears in regular smokers by sex adjusted for age, calendar year, ethnicity (first 40 principal components), height, recruitment centre (Model 1) and additionally for weight (Model 2). Non-linear relationships were captured using restricted cubic spline transformation with three knots placed at the 10th, 50th and 90th percentiles of urate level

Observational associations with urate differed by sex and smoking status. There was a weak U-shaped association between observed urate and the incidence of lung cancer in women without strong evidence of multiplicative interactions (Fig. 1). In contrast, we found strong L-shaped relationships between observed urate levels and lung cancer incidence in current smoking men but weaker associations in other smoking categories (Fig. 1). Reverse causation does not fully explain these associations, which remained similar after changing the date of cohort entry to one and two years after the urate measurement (Additional file 1: Fig. S1). We found L-shaped associations with lung cancer for men and women who smoked regularly (at least 1 cigarette per day), and these were slightly attenuated after adjusting for several other variables (Fig. 2). We found continuous by continuous interactions with packyears of smoking and lung cancer where the highest predicted incidence was for men and women with the lowest urate and highest number of pack-years (Fig. 3). Adjusted FEV_1_ declined across most smoking strata as observed levels of urate increased (Additional file 1: Fig. S2). Although for male current smokers, FEV_1_ increased up to around 300 µmol/L of urate followed by general decline (Additional file 1: Fig. S2). FEV_1_ declined as urate increased in current or former regular smokers and remained unchanged after adjustment (Fig. 3). There was evidence of similar interactions between FEV_1_ and pack-years with the lowest predicted FEV_1_ for men and women with the lowest urate and highest number of pack-years (Additional file 1: Fig. S3).

We confirmed that the selected SNPs were associated with urate levels explaining 5.3% (F statistic = 528) of the variability (Additional file 1: Fig. S4). The one-sample MR-analysis 376,922 participants had complete data (directly genotyped or imputed) for the two main SNPs and 305,614 had complete data for all 31 SNPs. There was no clear pattern in the per allele effects on FEV_1_ and lung cancer (Additional file 1: Fig. S4). The results of the MR with all 31 SNPs (Table 3) or with the two lead GWAS SNPs (Additional file 1: Table S1) did not support a causal association between urate and FEV_1_ or lung cancer. Separate analyses by sex and including probability weights for the analysis of FEV_1_ did not alter our overall conclusions. There was no evidence of an association between genetically predicted urate and a family history of lung cancer or prevalent COPD/emphysema (Additional file 1: Table S2).

**Table 3:** Associations between genetically predicted urate, baseline FEV1 and lung cancer incidence

	Coefficient per 100 µmol serum urate*	p value	Coefficient per 100 µmol serum urate*	p value	Incidence change per 10,000 PYs
	FEV_1_ (ml)		IRR lung cancer		
Overall	− 10.5 (− 23 to 2.1)	0.10	0.88 (0.68–1.14)	0.33	− 0.98 (− 2.95 to 1.00)
Never	− 12.3 (− 28.5 to 3.9)	0.14	0.88 (0.42–1.85)	0.74	− 0.25 (− 1.76 to 1.26)
Former	− 12.0 (− 32.9 to 8.9)	0.26	0.86 (0.59–1.26)	0.43	− 1.36 (− 4.75 to 2.03)
Current	11.0 (− 32.9 to 55.0)	0.62	0.91 (0.61–1.34)	0.62	− 3.40 (− 17.01 to 10.21)
Overall regular	− 5.0 (− 28.9 to 18.8)	0.68	0.88 (0.66–1.18)	0.40	− 2.43 (− 8.09 to 3.24)
Light former	− 14.3 (− 50.7 to 22.2)	0.44	1.60 (0.69–3.70)	0.27	3.63 (− 2.83 to 10.08)
Heavy former	− 17.6 (− 54.0 to 18.8)	0.34	0.68 (0.42–1.10)	0.12	− 6.11 (− 13.73 to 1.52)
Current light	15.9 (− 43.5 to 75.3)	0.60	1.00 (0.54–1.86)	1.00	0.05 (− 23.32 to 23.43)
Current heavy	40.9 (− 37.7 to 119.4)	0.31	0.56 (0.31–1.00)	0.05	− 39.10 (− 78.36 to 0.16)

*IRR* incidence rate ratio

*Adjusted for sex, age, calendar year, ethnicity (first 40 principal components) and recruitment centre

## Discussion

### Summary

As far as we know, this is the largest study to examine observational and potentially causal interactions between serum urate, cigarette smoking and lung cancer. Although the incidence of lung cancer was higher at the lowest levels of urate for men and women with a history of smoking and particularly for those with the highest number of pack-years, we found no substantial evidence to support causality. The observational associations, in our view, reflect residual confounding by factors associated with weight or diet. However, as a low cost and simple assay, the finding that associations remained after adjusting for several variables used in lung cancer risk prediction, suggests further work is needed to establish the value of urate in improving risk stratification. Low-dose computed tomography (CT) screening programmes are being adopted in the United States and piloted in the United Kingdom [27]. Even small improvements in risk prediction could have a meaningful impact due to the high mortality burden of lung cancer together with the financial and psychological cost of false-positives of CT-screening.

### Comparison with other studies

We found that higher levels of urate were independently associated with lower levels of FEV_1_, which is consistent with earlier findings for lung function in healthy people and for exacerbations and mortality associated with a COPD diagnosis [28–30]. In the absence of any strong indication of causality, the higher levels of urate in people with worse FEV_1_ could reflect reverse causation due to the cross-sectional design. For example, tissue hypoxia and inflammation can induce urate production by the degradation of adenosine triphosphate. Residual confounding due to omission or mismeasurement of causal variables could also explain the negative association between urate and FEV_1_. We found L-shaped associations between urate and lung cancer incidence in current and regular smokers with the predicted incidence highest in those with the greatest number of pack-years of smoking. A case-cohort study of urate and cancer reported negative associations with breast and cancer mortality, and weak negative trends for lung cancer that substantially weakened after adjustment [31]. No interactions were found with other variables including smoking status, although the smaller number of cases (n = 195) may have reduced precision of these estimates.

A cross-sectional study reported improved FEV_1_ in post-menopausal women with the *SLC2A9* variant (rs11722228) variant associated with raised urate, suggesting a role for female hormones in urate antioxidant activity [32]. In contrast, a recent phenome-wide association study (PheWAS) in UK biobank indicated unspecified diseases of the respiratory system were potentially causally increased in older women with genetically raised urate [33]. A large MR found no support for a causal association between genetically raised urate with lung function, higher risk of respiratory symptoms or COPD [34]. There were no clear interactions with sex and smoking status. A comprehensive review of hundreds of studies of urate including meta-analyses of observational, MR and randomised controlled trials, concluded there was only robust evidence of a positive association with gout and nephrolithiasis [35]. Our results for urate contrast with our recent findings for another endogenous antioxidant bilirubin [15]. In this case we found evidence of a causal relationship with lung cancer that increased in strength with smoking exposure.

### Strengths and limitations

The strengths of the present study are the large sample size, the longitudinal analysis for lung cancer and the availability of data on many potential confounders. The limitations include the use of self-report for smoking status, the short length of follow-up and potential for selection bias. UK Biobank participants are healthier compared to the wider population and the rates of smoking-related diseases, in particular, are substantially lower, which could lead to selection bias [36]. For example, suppose people with genetically low urate are less likely to recruited into UK Biobank due to poor health or death. In that case, the result could be an underestimation of the observational and causal associations. The selected genetic variants explained 5% of the variance in urate, which may have been too low to calculate precise causal estimates for lung cancer. Even larger cohorts than UK Biobank with a younger age at recruitment are needed to robustly exclude a causal association with lung cancer. However, we also found no supporting evidence of a causal relationship with the continuous outcome FEV_1_, which we might expect if urate is an important antioxidant. Although we found no strong evidence of a causal association with urate present in blood serum, this does not exclude an antioxidant role for urate found at high levels in the respiratory lining fluid.

## Conclusions

Self-reported current/regular smokers participating in UK Biobank with low levels of serum urate had higher rates of lung cancer and higher FEV_1_. Our findings using the Mendelian randomization approach, taken together with the existing literature, suggest serum urate is unlikely to represent a modifiable risk factor relevant to adult respiratory health and disease.

## Supplementary Information

**Additional file 1: Figure S1.** Observational associations between serum urate and lung cancer incidence by smoking status and sex after adding one and two years between the urate test date and cohort entry. **Figure S2.** Observational associations between serum urate and FEV_1_ by sex adjusted for age, calendar year, ethnicity (first 40 principal components), height and recruitment centre (Model 1) and additionally for weight (Model 2). Non-linear relationships were captured using restricted cubic spline transformation with three knots placed at the 10th, 50th and 90th percentiles of urate levels. **Figure S3.** Observational associations between serum urate and FEV_1_ with interactions with smoking packyears in regular smokers by sex adjusted for age, calendar year, ethnicity (first 40 principal components), height, recruitment centre (Model 1) and additionally for weight (Model 2). **Figure S4.** Per allele effects on serum urate, baseline FEV_1_ and lung cancer incidence. **Table S1.** Association between genetically predicted urate using rs12498742 and rs2231142, FEV_1_ and lung cancer incidence. **Table S2.** The genetically instrumented cross-sectional relationships between urate and other outcomes

## Data Availability

The data that support the findings of this study are available from UK Biobank but restrictions apply to the availability of these data, which were used under license for the current study, and so are not publicly available. Data are however available from the authors upon reasonable request and with permission of UK Biobank.

## Abbreviations

FEV_1_: Forced expiratory volume per second
IRR: Incidence rate ratio
MR: Mendelian randomization
PY: Person years
