# Supplementary material for: Serum urate and lung cancer: a cohort study and Mendelian randomization using UK Biobank
Source: Respir Res. 2021 Jun 16;22:179. doi: 10.1186/s12931-021-01768-y (PMC8210393; doi:10.1186/s12931-021-01768-y)
Supplement: Supplementary file 1 — Additional file 1: Figure S1. Observational associations between serum urate and lung cancer incidence by smoking status and sex after adding one and two years between the urate test date and cohort entry. Figure S2. Observational associations between serum urate and FEV1 by sex adjusted for age, calendar year, ethnicity (first 40 principal components), height and recruitment centre (Model 1) and additionally for weight (Model 2). Non-linear relationships were captured using restricted cubic spline transformation with three knots placed at the 10th, 50th and 90th percentiles of urate levels. Figure S3. Observational associations between serum urate and FEV1 with interactions with smoking packyears in regular smokers by sex adjusted for age, calendar year, ethnicity (first 40 principal components), height, recruitment centre (Model 1) and additionally for weight (Model 2). Figure S4. Per allele effects on serum urate, baseline FEV1 and lung cancer incidence. Table S1. Association between genetically predicted urate using rs12498742 and rs2231142, FEV1 and lung cancer incidence. Table S2. The genetically instrumented cross-sectional relationships between urate and other outcomes [file 12931_2021_1768_MOESM1_ESM.docx]

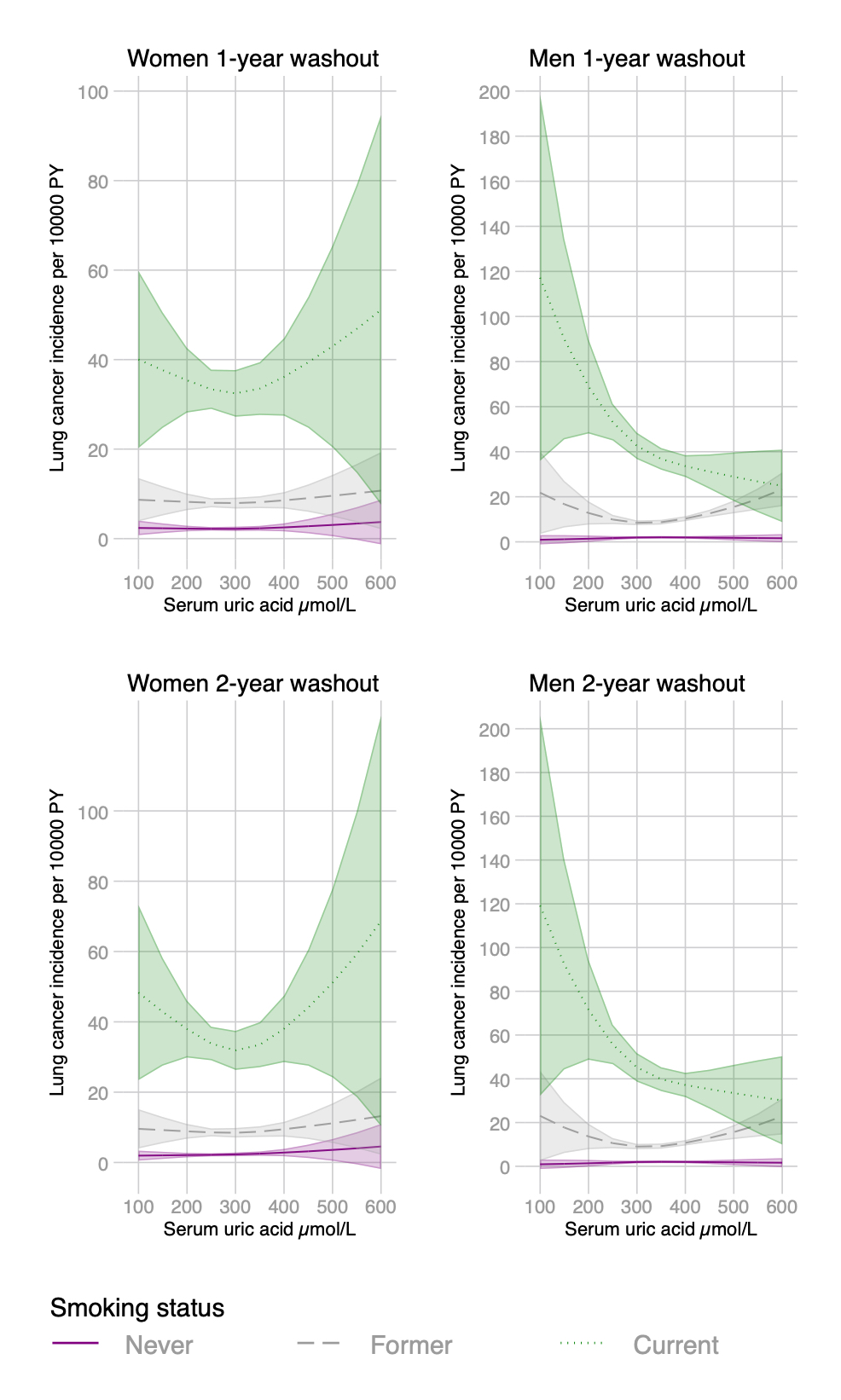


Figure S1: Observational associations between serum urate and lung cancer incidence by smoking status and sex after adding one and two years between the urate test date and cohort entry. Predicted incidence rates are adjusted for age, calendar year, ethnicity (first 40 principal components), height, weight and recruitment centre. Non-linear relationships were captured using restricted cubic spline transformation with three knots placed at the 10th, 50th and 90th percentiles of urate levels.


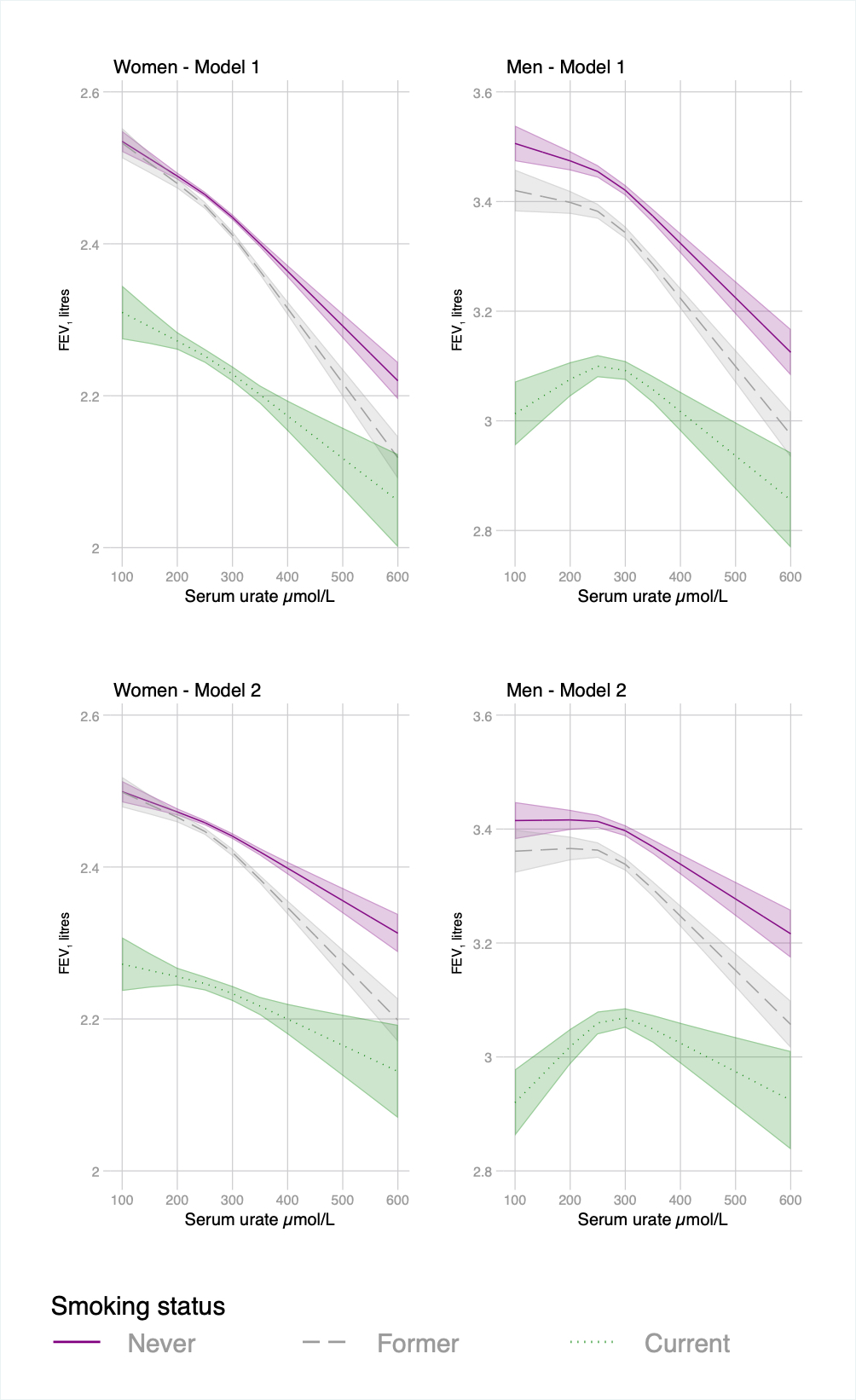


Figure S2: Observational associations between serum urate an d FEV_1_ by sex adjusted for age, calendar year, ethnicity (first 40 principal components), height and recruitment centre (Model 1) and additionally for weight (Model 2). Non-linear relationships were captured using restricted cubic spline transformation with three knots placed at the 10^th^, 50^th^ and 90^th^ percentiles of urate levels.

Table S1: Association between genetically predicted urate using rs12498742 and rs2231142, FEV_1_ and lung cancer incidence.

|  | Coefficient per 100 µmol/L* |  | Coefficient per 100 µmol/L* |  |  |
| --- | --- | --- | --- | --- | --- |
|  | FEV_1_ (ml) | p-value | IRR lung cancer | p-value | Incidence change per 10,000 PYs |
| Overall | -2.2 (-15.5 to 11.1) | 0.74 | -0.48 (-2.58 to 1.62) | 0.65 | 0.94 (0.71 to 1.24) |
| Never | 1.1 (-16.0 to 18.3) | 0.90 | 1.09 (0.47 to 2.52) | 0.84 | 0.17 (-1.53 to 1.88) |
| Former | -12.4 (-35.0 to 10.2) | 0.28 | 0.82 (0.55 to 1.21) | 0.31 | -1.77 (-5.22 to 1.68) |
| Current | 17.7 (-29.2 to 64.6) | 0.46 | 1.04 (0.67 to 1.59) | 0.87 | 1.28 (-13.67 to 16.22) |
|  |  |  |  |  |  |
| Overall regular | 4.6 (-21.4 to 30.5) | 0.73 | 0.96 (0.70 to 1.32) | 0.81 | -0.73 (-6.69 to 5.24) |
| Light former | -26.9 (-65.9 to 12) | 0.17 | 1.74 (0.70 to 4.36) | 0.24 | 4.15 (-2.73 to 11.02) |
| Heavy former | 15.6 (-25.6 to 56.8) | 0.46 | 0.71 (0.44 to 1.15) | 0.17 | -5.34 (-12.93 to 2.25) |
| Current light | 24.4 (-38.9 to 87.7) | 0.45 | 1.12 (0.55 to 2.26) | 0.75 | 3.95 (-20.8 to 28.7) |
| Current heavy | 60.6 (-35.6 to 156.7) | 0.22 | 0.70 (0.38 to 1.30) | 0.26 | -25.21 (-68.87 to 18.45) |

*Adjusted for sex, age, calendar year, ethnicity (first 40 principal components) and recruitment centre.

IRR=incidence rate ratio


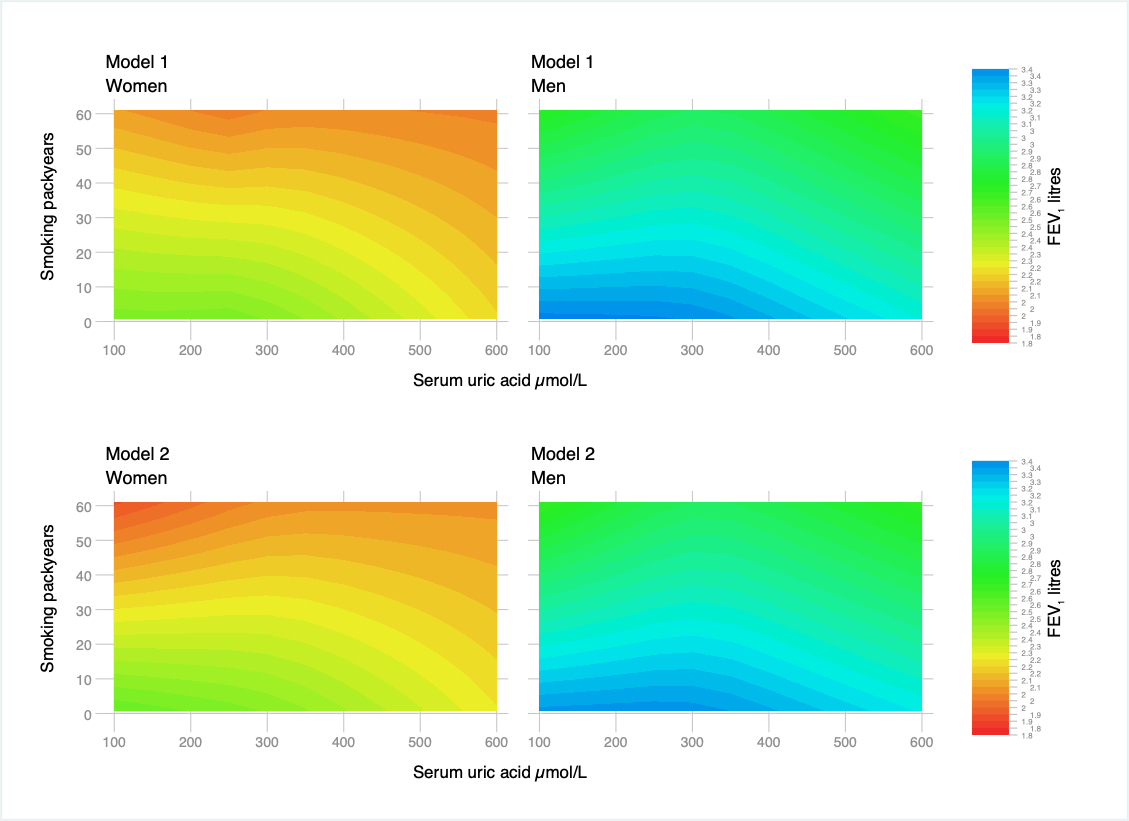


Figure S3: Observational associations between serum urate and FEV_1_ with interactions with smoking packyears in regular smokers by sex adjusted for age, calendar year, ethnicity (first 40 principal components), height, recruitment centre (Model 1) and additionally for weight (Model 2). Non-linear relationships were captured using restricted cubic spline transformation with three knots placed at the 10^th^, 50^th^ and 90^th^ percentiles of urate level.


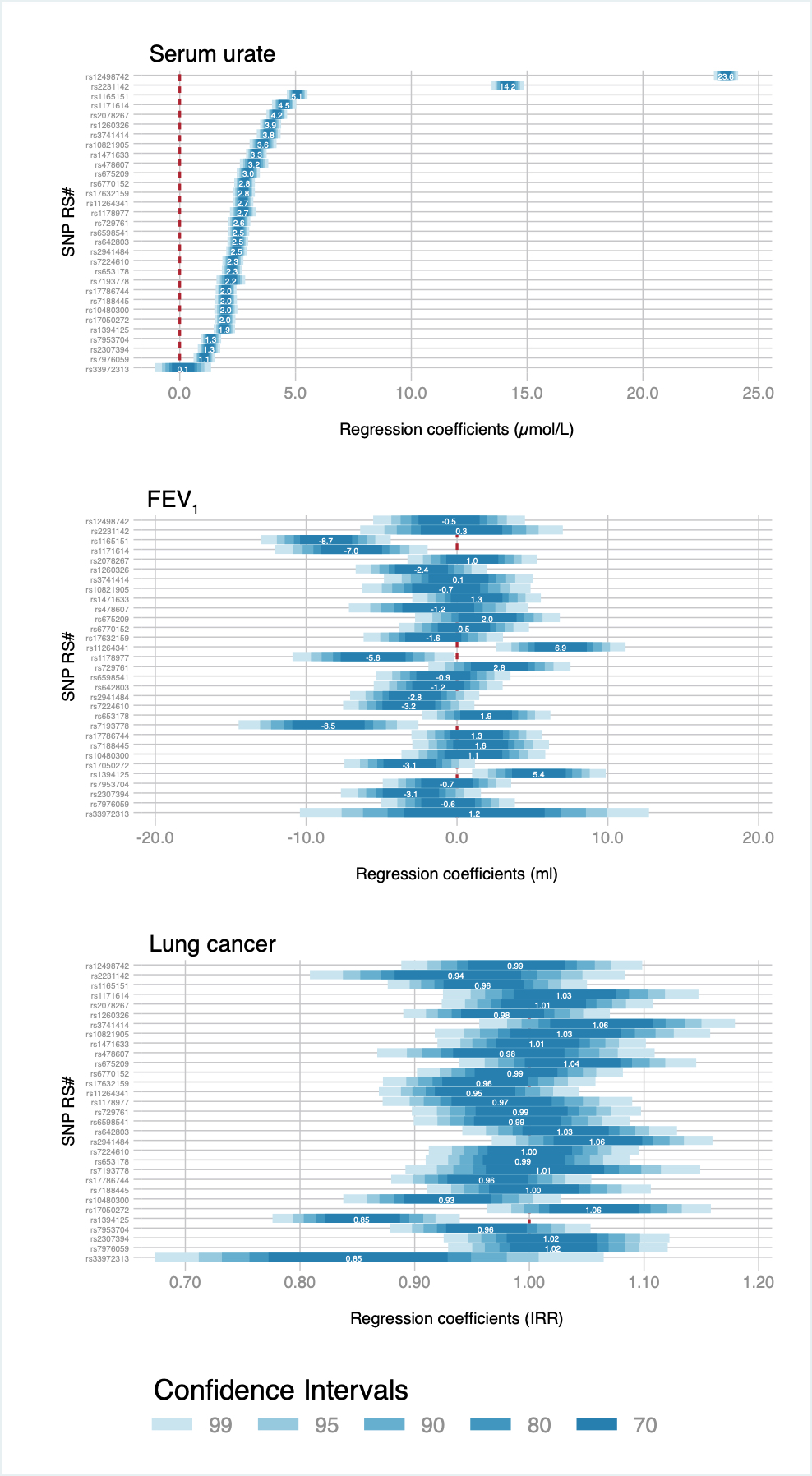


Figure S4: Per allele effects on serum urate, baseline FEV_1_ and lung cancer incidence.

Table S2: The genetically instrumented cross-sectional relationships between urate and other outcomes.

| Total | 305,614 | OR (95%CI) per 100 μmol/L increase* | p-value |
| --- | --- | --- | --- |
| Family history of lung cancer |  |  |  |
| Overall | 39,414 (12.9%) | 0.96 (0.90 to 1.01) | 0.13 |
| Never smokers | 20,521 (12.4%) | 0.95 (0.88 to 1.03) | 0.19 |
| Former smokers | 14,437 (13.4%) | 0.96 (0.88 to 1.06) | 0.43 |
| Current smokers | 4,294 (13.9%) | 0.99 (0.84 to 1.17) | 0.91 |
|  |  |  |  |
| History of COPD/emphysema |  |  |  |
| Overall | 6,979 (2.3%) | 0.99 (0.88 to 1.13) | 0.93 |
| Never smokers | 1,962 (1.1%) | 1.03 (0.81 to 1.29) | 0.83 |
| Former smokers | 3,377 (3.1%) | 0.94 (0.78 to 1.13) | 0.51 |
| Current smokers | 1,596 (5.1%) | 1.11 (0.85 to 1.45) | 0.42 |

*Adjusted for age, gender, calendar year, ethnicity (first 40 principal components) recruitment centre. Estimates derived using a one-sample Mendelian randomisation approach and the two‐stage predictor substitution method. Number may differ overall and by strata due to missing smoking data.
